# Non-Hermitian dynamics and non-reciprocity of optically coupled nanoparticles

**DOI:** 10.1038/s41567-024-02589-8

**Published:** 2024-07-25

**Authors:** Manuel Reisenbauer, Henning Rudolph, Livia Egyed, Klaus Hornberger, Anton V. Zasedatelev, Murad Abuzarli, Benjamin A. Stickler, Uroš Delić

**Affiliations:** 1 Vienna Center for Quantum Science and Technology, Faculty of Physics, University of Vienna, Vienna, Austria; 2 Faculty of Physics, University of Duisburg-Essen, Duisburg, Germany; 3 Institute for Complex Quantum Systems, Ulm University, Ulm, Germany

**Keywords:** Optomechanics, Nonlinear phenomena

## Abstract

Non-Hermitian dynamics, as observed in photonic, atomic, electrical and optomechanical platforms, holds great potential for sensing applications and signal processing. Recently, fully tuneable non-reciprocal optical interaction has been demonstrated between levitated nanoparticles. Here we use this tunability to investigate the collective non-Hermitian dynamics of two non-reciprocally and nonlinearly interacting nanoparticles. We observe parity–time symmetry breaking and, for sufficiently strong coupling, a collective mechanical lasing transition in which the particles move along stable limit cycles. This work opens up a research avenue of non-equilibrium multi-particle collective effects, tailored by the dynamic control of individual sites in a tweezer array.

## Abstract (web summary)

The tuneable and nonlinear nature of the interactions between two optically levitated nanoparticles allows the observation of the system’s non-Hermitian dynamics and a mechanical lasing transition.

## Main

A plethora of physical phenomena are well described by Hermitian dynamics, such as the dynamics of closed quantum systems, Landau-type phase transitions or transport along oscillator chains. However, the growth of complexity in quantum many-body systems, the occurrence of chiral transport properties and the emergence of experiments that strongly couple to the environment require models that go beyond Hermitian descriptions. A particularly interesting example of this so-called non-Hermitian dynamics is non-reciprocal interactions, which seemingly break Newton’s third law of action equals reaction. Besides being often encountered in biological systems^1^, non-reciprocity—in its broadest sense—has found various applications in optics and photonics^2–8^, ultracold atoms^9–15^, electrical circuits^16,17^ and metamaterials^18–21^. The size and sensitivity to environmental perturbations make non-reciprocally interacting arrays of mechanical objects an ideal ground for realizing unidirectional or topological transport^22–24^, enhanced sensing due to non-reciprocity^25–27^, and topological states^28–30^. So far, there have been several experimental demonstrations of non-reciprocal or non-Hermitian dynamics in various platforms in the classical regime^31–38^.

Optically levitated nanoparticles have become a well-established system for quantum physics with translational and rotational degrees of freedom^39,40^. Recently, there has been a surge of experiments that extend trapping and control from single particles to particle arrays in a variety of geometries^41–46^, thus demonstrating that this platform is highly versatile and scalable. In one of those experiments, we have demonstrated direct, non-reciprocal and nonlinear light-induced dipole–dipole interactions between particles in a tweezer array^41^. Such optically interacting particle arrays offer several benefits for investigations of (quantum) non-Hermitian physics. For example, single-site readout enables the full reconstruction of the collective degrees of freedom^44,46,47^. At the same time, the optically induced forces allow for a wide tuning range from reciprocal to unidirectional to anti-reciprocal interactions^48^. Altogether, this system enables studies in previously unexplored interaction regimes with an unprecedented level of control.

In this work, we report the experimental investigation of non-Hermitian dynamics stemming from the anti-reciprocal and nonlinear interaction between the motion of two trapped silica nanoparticles. We observe two exceptional points (EP) that define a region where the collective motion is in the parity and time-reversal (${{{\mathcal{PT}}}}$) symmetry-broken phase. In this phase, the interaction leads to correlated particle motion, which we confirm by measuring a constant phase delay between the oscillators. The system further exhibits a Hopf bifurcation into the mechanical lasing phase, where the interaction-induced amplification dominates over the intrinsic damping such that the motion becomes nonlinear.

## Experimental setup

**Fig. 1:**
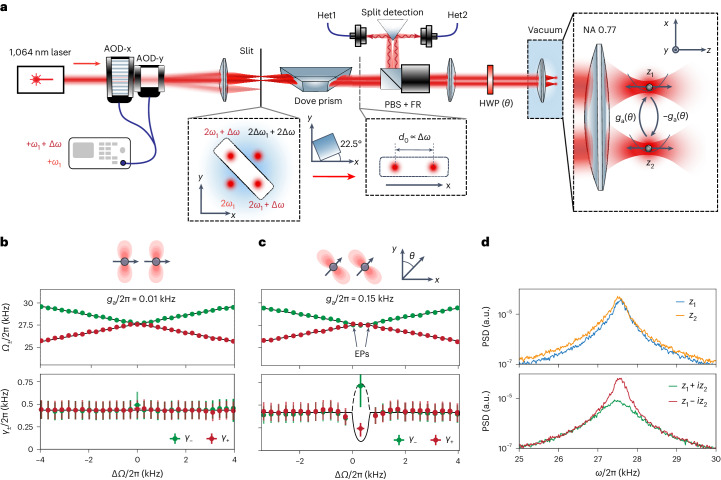
Experimental setup. **a**, Each of the two orthogonal AODs (AOD-x/y) is driven by the same two RF tones at frequencies *ω*_1_ and *ω*_1_ + Δ*ω*, which creates 2 × 2 laser beams. We use a slit to select the two beams that have equal optical frequencies, while the distance between the beams can be tuned by changing the RF tone frequency difference Δ*ω*. The Dove prism rotates the optical plane to place the beams into the plane of the optical table. The beams are subsequently focused in the vacuum chamber to form two traps at a distance *d*_0_. The light back-scattered by the particles is reflected with a Faraday rotator (FR) and a polarizing beamsplitter (PBS) and sent to two independent heterodyne detectors monitoring particle motion. Inset: two particles are trapped and interact anti-reciprocally with the coupling rate ± *g*_a_ tuned by the polarization angle *θ*, which we set with a HWP in front of the vacuum chamber. **b**, For polarization along the *x* axis (*θ* ≈ π/2), the interaction between the particles is weak such that the two modes cross. **c**, For *θ* ≠ π/2, we observe degenerate eigenfrequencies between the two EPs (top) and non-degenerate damping rates that are split by 4*g*_a_ (bottom). The black lines are theory functions based on the measured coupling rate. In **b** and **c**, the green and red points represent measured eigenfrequencies and damping rate extracted from fitted spectral peaks. The black lines are fits. The error bars correspond to the standard deviation error of the fits. **d**, At the maximum splitting of the damping rates, we can reconstruct the PSDs of the eigenmodes of the particles' positions as *z*_1_ ± *iz*_2_ (bottom) from the detected positions *z*_1,2_ (top).

We use a pair of orthogonal acousto-optical deflectors (AODs), both driven by two radiofrequency (RF) tones at frequencies *ω*_1_ and *ω*_1_ + Δ*ω*, to create 2 × 2 laser beams from a laser source at a wavelength of *λ* = 1,064 nm (Fig. 1a). We use a narrow slit (width 2 mm) to select the laser beams on the diagonal as they have equal laser frequencies, which is required to control the optical interaction. A Dove prism tilted at an angle of 22. 5° rotates the optical plane by 45°, thus placing the laser beams in the plane of the optical table. A lens with a high numerical aperture (NA, 0.77) inside the vacuum chamber focuses the laser beams to two foci at a relative distance *d*_0_, in which we trap two silica nanoparticles of approximately equal sizes (nominal radius *r* = (105 ± 2) nm; Methods). We tune the particle distance with the frequency difference of the RF tones as *d*_0_ ∝ Δ*ω* on both AODs, which maintains equal laser frequencies of the two tweezers (Methods). The optical phases at the traps *ϕ*_1,2_ are controlled by the phases of the RF tones. For optical powers of *P*_1,2_ ≈ 0.3 W the resulting oscillation frequencies of the centre-of-mass (CoM) motion along the tweezer axis (*z* axis) are ${{{\varOmega }}}_{1,2}\propto \sqrt{{P}_{1,2}}\approx 2\uppi \times 27.5\,{{{\rm{kHz}}}}$. To sweep the mechanical frequency detuning Δ*Ω* = *Ω*_2_ − *Ω*_1_, we scan powers *P*_1,2_ symmetrically such that the total power is conserved. We collect the light back-scattered from the particles with separate fibre-based confocal microscopes, which allows us to independently detect the particles’ motion with balanced heterodyne detections (Methods). To suppress electrostatic coupling, we place the particles at a large distance of *d*_0_ ≈ 18.4 μm and discharge them (Methods). The intrinsic damping rate, given by the gas pressure, is kept constant at *γ*/2π = (0.46 ± 0.02) kHz throughout the measurement.

## Non-Hermitian dynamics

We model the particles’ *z* motion in the frame rotating with the mean oscillation frequency *Ω*_0_ = (*Ω*_1_ + *Ω*_2_)/2. The linearized equations of motion, averaged over one oscillation period 2π/*Ω*_0_, are

1 $$\frac{{\mathrm{d}}}{{\mathrm{d}}t}\left(\begin{array}{r}{a}_{1}\\ {a}_{2}\\ \end{array}\right)=-i{H}_{{{{\rm{NH}}}}}\left(\begin{array}{r}{a}_{1}\\ {a}_{2}\\ \end{array}\right)+\sqrt{\gamma {n}_{{\mathrm{T}}}}\left(\begin{array}{r}{\xi }_{1}\\ {\xi }_{2}\\ \end{array}\right),$$

where ${a}_{j}=\left({z}_{j}+i{p}_{j}/m{{{\varOmega }}}_{j}\right)\exp (i{{{\varOmega }}}_{0}t)/2{z}_{{{{\rm{zpf}}}},\;j}$ is the complex amplitude of particle *j* with position *z*_*j*_, momentum *p*_*j*_, zero point fluctuation ${z}_{{{{\rm{zpf}}}},\;j}=\sqrt{\hslash /2m{{{\varOmega }}}_{j}}$ and mass *m*. Gas damping and diffusion enters via the gas damping rate *γ*, the thermal occupation *n*_T_ in equilibrium with the environment and the complex white noises *ξ*_1,2_ with correlations $\langle {\xi }_{j{\prime} }^{\;* }(t{\prime} ){\xi }_{j}(t)\rangle ={\delta }_{jj{\prime} }\delta (t-t{\prime} )$ and $\langle {\xi }_{j{\prime} }(t{\prime} ){\xi }_{j}(t)\rangle =0$. The effectively non-Hermitian dynamics realized by the optical interaction is generated by the matrix^48^

2 $${H}_{{{{\rm{NH}}}}}=\left(\begin{array}{ll}-\frac{{{\Delta }}{{\varOmega }}}{2}+{g}_{{\mathrm{r}}}+{g}_{{\mathrm{a}}}-i\frac{\gamma }{2}\qquad\quad-({g}_{{\mathrm{r}}}+{g}_{{\mathrm{a}}})\\\qquad\;-({g}_{{\mathrm{r}}}-{g}_{{\mathrm{a}}})\qquad\qquad\frac{{{\Delta }}{{\varOmega }}}{2}+{g}_{{\mathrm{r}}}-{g}_{{\mathrm{a}}}-i\frac{\gamma }{2}\\ \end{array}\right),$$

where ${g}_{{\mathrm{r}}}=G\cos (k{d}_{0})\cos ({{\Delta }}\phi )/k{d}_{0}$ and ${g}_{{\mathrm{a}}}=G\sin (k{d}_{0})\sin ({{\Delta }}\phi )/k{d}_{0}$, with *k* = 2π/*λ*, describe the reciprocal (conservative) and the anti-reciprocal (non-conservative) coupling rate, respectively^41,48^. The non-reciprocity of the effective interaction arises from the interference of the tweezer and the light scattered off the particles, which effectively carries away momentum. The interference and, thus, the coupling rates can be tuned by the optical phase difference Δ*ϕ* = *ϕ*_2_ − *ϕ*_1_ and the distance *d*_0_ between the particles. In our work, we set *d*_0_ and Δ*ϕ* such that *g*_r_ is negligible, while *g*_a_ takes its maximum value for a given distance. Its magnitude is then determined by the coupling constant $G\propto \sqrt{{P}_{1}{P}_{2}}{\cos }^{2}(\theta )$ as a function of the trapping powers *P*_1,2_ and the laser polarization angle *θ* (Fig. 1b,c), which we modify with a half-wave plate (HWP) in front of the vacuum chamber (Methods). The eigenvalues of *H*_NH_ are in general complex and are given by ${\lambda }_{\pm }=-i\gamma /2\pm \sqrt{{{\Delta }}{{{\varOmega }}}^{2}-4{g}_{{\mathrm{a}}}{{\Delta }}{{\varOmega }}}/2$, such that the frequencies and damping rates of the eigenmodes are given by the real and imaginary parts *Ω*_±_ = *Ω*_0_ + Re(*λ*_±_) and *γ*_±_ = −2Im(*λ*_±_), respectively. The eigenvectors coalesce at the detunings of Δ*Ω*_EP1,2_ = 2*g*_a_ ∓ 2*g*_a_, which define the EPs. For Δ*Ω* between the EPs, the frequencies are degenerate and the damping rates become non-degenerate, resulting in the so-called normal mode attraction (Fig. 1c). The maximum splitting of the damping rates *γ*_±_ is achieved for Δ*Ω* = 2*g*_a_ and is equal to 4*g*_a_, where the complex eigenmodes of the system can be reconstructed as *a*_±_ = *a*_1_ ± *ia*_2_. Note that the sign in the corresponding eigenmodes of the motion *z*_±_ = *z*_1_ ∓ *iz*_2_ is flipped due to the definition of *a*_1,2_. Therefore, the different damping rates result in the suppression of the eigenmode *a*_−_ (*z*_−_), while *a*_+_ (*z*_+_) is amplified (Fig. 1d). Note that *H*_NH_ is ${{{\mathcal{PT}}}}$-symmetric in a generalized sense^1^; however, this ${{{\mathcal{PT}}}}$ symmetry is broken in the region between the EPs as the eigenmodes have different damping rates (Methods).

## Nonlinear anti-reciprocal interactions

Once the effective damping rate *γ*_+_ becomes negative, the linear theory breaks down as the amplitude of the eigenmode *a*_+_ increases exponentially. In this case, the particle motion starts exploring the intrinsic nonlinearity of the optical binding forces, which act to stabilize the oscillation amplitude. This mechanism is in stark contrast to other systems where two parametric limit-cycle oscillators are coupled linearly, regardless of the reciprocal or non-reciprocal nature of their coupling mechanism^49–51^. An analytical model that includes the full nonlinear dynamics—but no thermal fluctuations—yields a system of differential equations for the amplitude of the collective motional state $A=2k\sqrt{\hslash /m{{{\varOmega }}}_{0}}\sqrt{| {a}_{2}{| }^{2}+| {a}_{1}{| }^{2}}$ and the phase delay between the oscillators $\psi =\arg [{a}_{2}^{* }{a}_{1}]$ (Methods):

3 $$\begin{array}{lll}\dot{A}&=&-\frac{\gamma }{2}A+{g}_{{\mathrm{a}}}A\sin (\psi )f\left(A\sin \frac{\psi }{2}\right),\\ \dot{\psi }&=&{{\Delta }}{{\varOmega }}-4{g}_{{\mathrm{a}}}{\sin }^{2}\left(\frac{\psi }{2}\right)f\left(A\sin \frac{\psi }{2}\right).\end{array}$$

Here, *f*(*x*) = 2*J*_1_(*x*)/*x* depends on the first-order Bessel function of the first kind *J*_1_. The equations of motion (3) are valid only in the ${{{\mathcal{PT}}}}$ symmetry-broken phase and have two steady-state solutions: (1) a collective state with vanishing oscillations (*A* = 0) but a stable phase delay $\psi =2\arcsin (\sqrt{{{\Delta }}{{\varOmega }}/4{g}_{{\mathrm{a}}}})$ and (2) a coherently oscillating state with *A* satisfying $f\left(A{{\Delta }}{{\varOmega }}/\sqrt{{\gamma }^{2}+{{\Delta }}{{{\varOmega }}}^{2}}\right)=({\gamma }^{2}+{{\Delta }}{{{\varOmega }}}^{2})/4{g}_{{\mathrm{a}}}{{\Delta }}{{\varOmega }}$ and phase delay $\psi =2\arctan ({{\Delta }}{{\varOmega }}/\gamma )$. As *f*(*x*) ≤ 1, the two solutions exist in regions separated by the threshold defined by ${g}_{{\mathrm{a}}}=\left({\gamma }^{2}+{{\Delta }}{{{\varOmega }}}^{2}\right)/4{{\Delta }}{{\varOmega }}$. Above this threshold, the first solution becomes unstable and the second, truly nonlinear solution—a stable limit cycle—emerges, thus revealing a Hopf bifurcation.

**Fig. 2:**
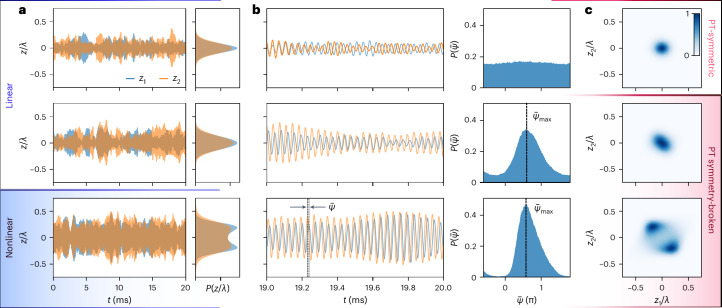
Collective particle motion. The time traces and statistics of the particles’ position *z*_1_ (blue) and *z*_2_ (orange) differ in the weakly coupled (*g*_a_/*γ* ≈ 0.01 and Δ*Ω*/*γ* ≈ 1.18, top row), linear (*g*_a_/*γ* ≈ 0.32 and Δ*Ω*/*γ* ≈ 1.19, middle row) and nonlinear regime (*g*_a_/*γ* ≈ 1.04 and Δ*Ω*/*γ* ≈ 1.37, bottom row). In the middle and bottom rows, the system is in the ${{{\mathcal{PT}}}}$ symmetry-broken phase. **a**, The histograms of the particles’ motion show an increasing variance (top to middle) and eventually the transition from linear into nonlinear motion (bottom). **b**, Uncoupled particles move independently, which is confirmed by the uniform distribution of the phase delay $\bar{\psi }$ between the oscillators. On the other hand, in the ${{{\mathcal{PT}}}}$ symmetry-broken phase the histograms show a preferred phase ${\bar{\psi }}_{\max }$ as *z*_1_ and *z*_2_ are strongly correlated. The black lines mark the most probable phase delay ${\bar{\psi }}_{\max }$. **c**, The joint *z*_1_–*z*_2_ distributions show the transition from a thermal motion (top) to a correlated motion (middle) to a limit cycle (bottom).

The dynamics of the collective motion under conditions defined by the two solutions of equation (3) are shown in the middle (*g*_a_/*γ* = 0.32 ± 0.05, Δ*Ω*/*γ* = 1.19 ± 0.05) and bottom row (*g*_a_/*γ* = 1.04 ± 0.08, Δ*Ω*/*γ* = 1.37 ± 0.06) of Fig. 2, respectively, while the top row features the standard behaviour of weakly coupled (*g*_a_/*γ* = −0.01 ± 0.03, Δ*Ω*/*γ* = 1.18 ± 0.05) thermal oscillators for comparison. In the linear regime, the individual particles’ motion follows a Gaussian distribution, and we observe an increased motional amplitude as we transition into the ${{{\mathcal{PT}}}}$ symmetry-broken phase (Fig. 2a). For higher coupling rates, the particles’ motion becomes nonlinear, which is reflected in modified motional statistics to a displaced Gaussian distribution. We compute the phases ${\bar{\psi }}_{1,2}$ of the individual particles’ motion via the Hilbert transform and calculate the instantaneous phase delay between the oscillators as $\bar{\psi }={\bar{\psi }}_{2}-{\bar{\psi }}_{1}$ (Methods). The stable phase delay $\bar{\psi }$ is resolved in the zoomed-in time trace of the nonlinear motion (Fig. 2b). The phase histogram is almost perfectly uniform for weakly coupled oscillators in the ${{{\mathcal{PT}}}}$-symmetric region, where the residual correlation arises from thermal fluctuations (Methods). On the other hand, the interaction generates a strongly preferred phase delay ${\bar{\psi }}_{\max }$ as observed in the histograms of $\bar{\psi }$ in the ${{{\mathcal{PT}}}}$ symmetry-broken phase. The *z*_1_–*z*_2_ distribution shows the collective dynamics of the two particles (Fig. 2c). In the case of weakly coupled particles, the distribution is well described by an uncorrelated two-dimensional Gaussian distribution. However, in the case of solution 1, *z*_1_ and *z*_2_ are strongly correlated and, thus, the joint distribution is squashed under an angle that depends on $\bar{\psi }$. For nonlinear motion, the *z*_1_–*z*_2_ distribution exhibits a stable path—the limit cycle.

**Fig. 3:**
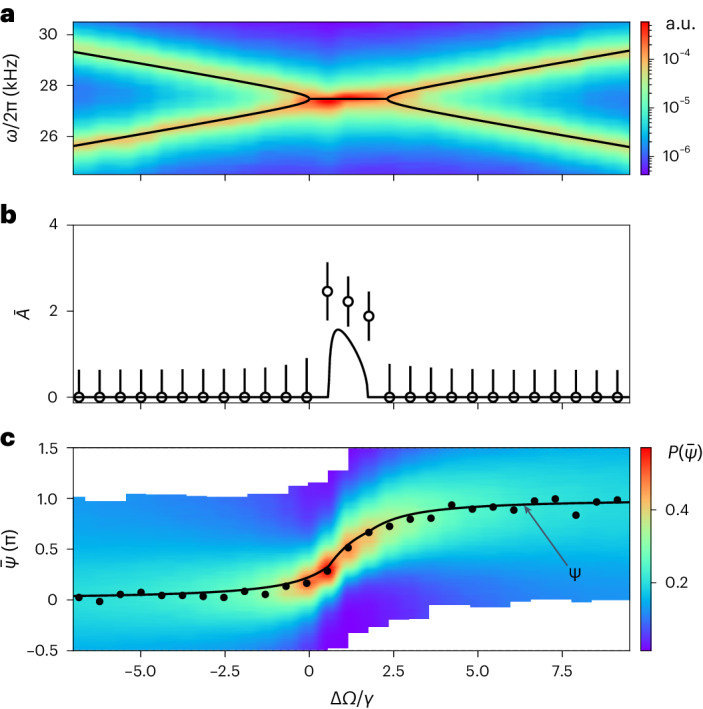
Amplitude and phase delay of nonlinear motion. **a**, The spectrogram of the particle motion reveals the mode eigenfrequencies as a function of Δ*Ω*/*γ*, where from the fit (black lines) we obtain a coupling of *g*_a_/*γ* = 0.58 ± 0.05. The frequencies are degenerate for 0 < Δ*Ω*/*γ* ≲ 2.3. **b**, Displacement amplitude $\bar{A}$ (points) extracted from fits as described in the main text. The error bars represent the standard error of the fit. The line shows theoretical limit cycle amplitude *A* as a function of Δ*Ω*/*γ*. The displacement amplitude becomes non-zero within the ${{{\mathcal{PT}}}}$ symmetry-broken phase whenever the particles’ motion are limit cycles. **c**, The probability density of the phase delay $\bar{\psi }$ is shown in colour, where the black points show the obtained ${\bar{\psi }}_{\max }$ at each detuning Δ*Ω*/*γ*. For large detunings ∣Δ*Ω*/*γ*∣ ≫ 0, histograms of $\bar{\psi }$ follow an approximately uniform distribution. Within the ${{{\mathcal{PT}}}}$ symmetry-broken phase, $\bar{\psi }$ acquires a strongly preferred value that depends on Δ*Ω*. The solid black line is the theory plot that combines the linear and nonlinear models for *ψ*.

We compare the theoretical steady-state solutions *A* and *ψ* to the measured displacement amplitude $\bar{A}$ and phase delay $\bar{\psi }$ as a function of the mechanical detuning Δ*Ω*/*γ* (Fig. 3). We obtain the coupling rate of *g*_a_/*γ* = 0.58 ± 0.05 from the fit of the eigenfrequencies *Ω*_±_ to the spectrogram of particle motion (Fig. 3a). The region between the EPs, given by 0 ≤ Δ*Ω*/*γ* ≲ 2.3, defines the ${{{\mathcal{PT}}}}$ symmetry-broken phase. To obtain the displacement amplitude $\bar{A}$ from the particle motion *z*_1,2_, we first reconstruct the complex amplitudes *a*_1,2_ (Methods). We model the histograms of ∣*a*_1,2_∣ with Rice distributions and fit the individual displacements ${\bar{A}}_{1,2}$, which we use to calculate $\bar{A}=2k\sqrt{\hslash /m{{{\varOmega }}}_{0}}\sqrt{| {\bar{A}}_{2}{| }^{2}+| {\bar{A}}_{1}{| }^{2}}$. Although there is a good agreement between the predicted *A* (line) and reconstructed amplitudes $\bar{A}$ (points) (Fig. 3b), we attribute the discrepancy between them to thermal fluctuations that are not included in our theory model. As the phase is periodic with 2π, we plot the histograms of $\bar{\psi }\,{{\mathrm{mod}}}\,\,2\uppi$ (coloured density plot), centred around the preferred phase ${\bar{\psi }}_{\max }$ (black points), as a function of Δ*Ω*/*γ* (Fig. 3c). Throughout the scan, the phase difference undergoes a π shift, as predicted by our model (black line) that combines the nonlinear theory in the range where *A* ≠ 0 and the full linear theory with included thermal fluctuations elsewhere.

## Mechanical lasing transition

**Fig. 4:**
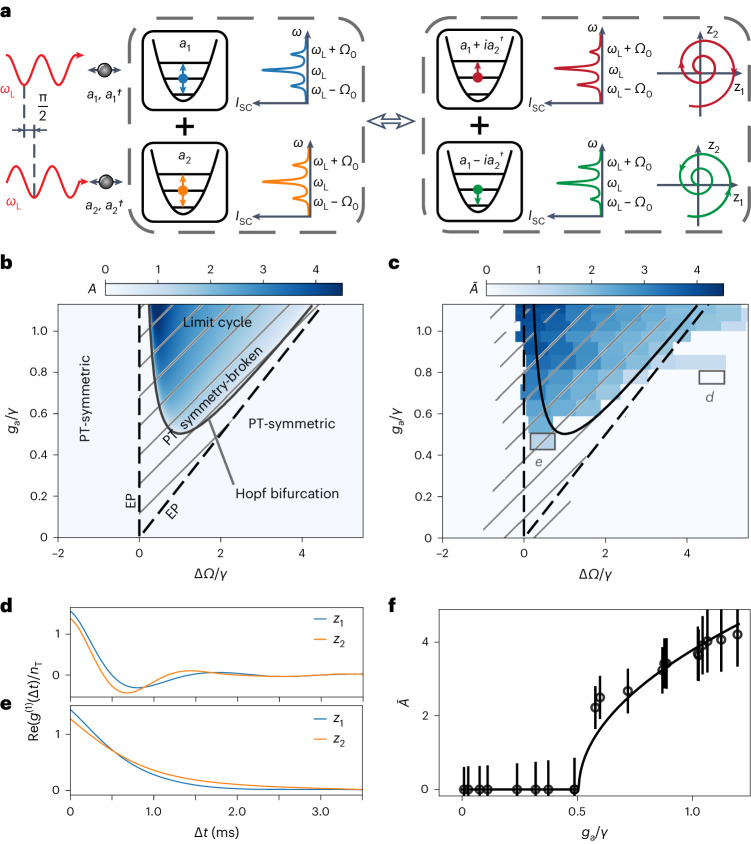
Non-Hermitian phases. **a**, The motion of particles 1 (blue) and 2 (orange) along the optical axes generates Stokes and anti-Stokes sidebands at *ω*_L_ − *Ω*_0_ and *ω*_L_ + *Ω*_0_, respectively, from the intrinsic laser frequency *ω*_L_. The optical interaction leads to modified amplitudes of Stokes and anti-Stokes sidebands of the eigenmodes *a*_1_ − *ia*_2_ (red) and *a*_1_ + *ia*_2_ (green), amplifying and damping the modes with suppressed anti-Stokes (red) and Stokes sideband (green), respectively. **b**, Expected amplitude *A* of the limit cycle as a function of the detuning Δ*Ω*/*γ* and the anti-reciprocal coupling *g*_a_/*γ*. The oscillators are in the ${{{\mathcal{PT}}}}$ symmetry-broken phase (hatched region) between the EPs (dashed lines). Outside of this region, the oscillators are in the ${{{\mathcal{PT}}}}$-symmetric phase. The black line marks the Hopf bifurcation of the nonlinear model. The collective motion follows the limit cycles in regions with non-zero amplitude *A* (shades of blue). **c**, Measured displacement amplitude $\bar{A}$, where each pixel represents a single measurement with its lower border defined by the coupling rate and its centre given by the detuning. **d**,**e**, The autocorrelation function *g*^(1)^(Δ*t*) for the parameters marked in **c** shows oscillatory (**d**) (bi-exponential, **e**) behaviour in the ${{{\mathcal{PT}}}}$-symmetric (**d**) (symmetry-broken, **e**) phase. The shaded region in **b** marks the ${{{\mathcal{PT}}}}$ symmetry-broken phase if the measured autocorrelation function *g*^(1)^(Δ*t*) shows a bi-exponential decay. **f**, The region of the limit cycle solutions ($\bar{A} > 0$) occurs at a threshold coupling rate of *g*_a_/*γ* > 0.5 for Δ*Ω*/*γ* ≈ 0.9. The points represent the fitted $\bar{A}$ as described in the main text, with their error bars representing the standard error of the fit and the line representing a theory fit. We increase the coupling rate *g*_a_ by rotating the laser polarization from horizontal to vertical.

The Hopf bifurcation and the increase of the limit cycle amplitude *A* may be interpreted as a mechanical lasing transition. In analogy to a laser, here the two-level laser medium is represented by the highly populated tweezer (upper level) and the Stokes sideband given by the particle motion (lower level). Scattering of the tweezer mode into the Stokes sideband creates a phonon in the mechanical degrees of freedom of the particles. In past works, one or more cavities were required to suppress the opposite transition into the anti-Stokes sideband^52^. Instead of enhancing the Stokes scattering via the Purcell effect, here an interference between the mechanical sidebands created by the particles’ motion leads to a suppression of the Stokes or anti-Stokes scattering, depending on which mechanical mode is excited (Fig. 4a). This interference leads to an amplified (suppressed anti-Stokes scattering) and a decaying mechanical mode (suppressed Stokes scattering). The lasing threshold is reached when the amplification exceeds the net loss, given by the intrinsic damping of the mechanical modes.

To fully characterize the mechanical lasing transition, we repeat the measurements of $\bar{A}$ as a function of Δ*Ω*/*γ* for varying couplings *g*_a_/*γ*. Comparison of *A* from the nonlinear theory model in equation (3) (Fig. 4b) with the extracted $\bar{A}$ (Fig. 4c) shows a good agreement, which demonstrates that the observed nonlinear dynamics is well described by our theory model. We distinguish three regions in both graphs. For 0 < Δ*Ω*/*γ* < 4*g*_a_/*γ* (hatched area), the oscillators are in the ${{{\mathcal{PT}}}}$ symmetry-broken phase, while they are in the ${{{\mathcal{PT}}}}$-symmetric phase elsewhere. Each particle motion is a combination of the two eigenmodes, which have non-degenerate frequencies outside and non-degenerate damping rates inside the ${{{\mathcal{PT}}}}$ symmetry-broken phase. Therefore, the crossing between these phases is characterized by the change of the amplitude correlation functions ${g}_{jj}^{(1)}({{\Delta }}t)=\langle {a}_{j}^{* }(t){a}_{j}(t+{{\Delta }}t)\rangle$ from an oscillatory behaviour outside (Fig. 4d), to a bi-exponential decay inside the symmetry-broken phase (Fig. 4e). Furthermore, above the threshold marked by the solid black line, the oscillators exhibit the mechanical lasing transition of equation (3). To clearly show the transition, we plot $\bar{A}$ as a function of the coupling at a detuning of Δ*Ω*/*γ* ≈ 0.9 (Fig. 4f). A non-zero $\bar{A}$ emerges at the threshold coupling of *g*_a_/*γ* ≈ 0.5 and follows a square-root dependence on the coupling, as predicted by the theory. The mechanical lasing transition could be understood as a second-order (non-Hermitian) phase transition^1^, bearing in mind that our system does not exhibit an apparent thermodynamic limit.

## Conclusion

We investigated the collective linear and nonlinear dynamics of two anti-reciprocally coupled optically levitated nanoparticles, which interact through light-induced dipole–dipole forces. The particles’ motion was monitored independently, which allowed us to reconstruct the eigenmodes of the system and to observe signatures of the ${{{\mathcal{PT}}}}$ symmetry breaking. Within the ${{{\mathcal{PT}}}}$ symmetry-broken phase, two eigenmodes with different damping rates emerge and the particles’ motion become strongly correlated, which we confirm by measuring a stable phase delay between the oscillators. For a sufficiently high coupling rate, the system passes through a Hopf bifurcation where the joint phase space distribution exhibits a limit cycle.

The presented steady-state measurements are a first step towards probing effects arising from the dynamical operation of non-reciprocally coupled particle chains induced by, for example, encircling the EP for topological energy transfer^32,33,53^. Larger arrays of tuneable, non-reciprocally interacting optically levitated particles have a great potential for studies of non-reciprocal phase transitions^1^ and non-equilibrium physics^54–56^ and may have sensing applications^27,57–61^. A combination of long-range non-reciprocal interactions and the already demonstrated quantum control of linear particle motion^62^ or cooling of limit cycle fluctuations^63^ holds promise to be a game changer for studying collective quantum phenomena, such as non-Hermitian quantum physics with long-range interactions^64^, entanglement in an optical cavity and observation of quantum optical binding^48^. We are aware of related work by Liška et al.^65^.

## Methods

### Theoretical model

In this section, we discuss the derivation of the linear and nonlinear models (1) and (3) for the collective motion of the two trapped nanoparticles from the theory of optical interactions^48,66^. The starting point is the quantum Langevin equations from ref. ^48^ for light-induced dipole–dipole interactions between arbitrary mechanical degrees of freedom. We consider two identical spherical particles in their respective tweezer traps (see equation (69) in ref. ^48^), ignore quantum noise, treat all operators as classical variables and add gas damping and diffusion with damping constant *γ* and gas temperature *T*_g_. The tweezers are assumed to have identical (linear) polarizations and waists but may be driven with a different power. The mechanical frequencies along transverse directions are substantially higher than the frequency along the optical axis; therefore, we restrict the dynamics to the particle motion along the tweezer propagation direction.

To determine the main nonlinearity of the two-particle dynamics around the tweezer foci, we expand the optical potential and the non-conservative radiation pressure force to the first-order nonlinearity, while in the dipole–dipole interaction, the tweezer fields are well approximated by plane waves. Furthermore, the equations of motion are expanded to leading order in the inverse distance 1/*d*_0_ between the tweezer foci, since far-field coupling dominates.

The resulting equations of motion for the particle coordinates *z*_1,2_ and momenta *p*_1,2_ along the optical axis can be written in terms of the complex amplitudes *a*_1,2_, as defined below in equation (1). For the experiment described in this Article, the influences of gas collisions, nonlinearities in the optical potential, the non-conservative radiation pressure forces, dipole–dipole interactions and the mechanical frequency difference Δ*Ω* are weak during one cycle of the mean harmonic motion *Ω*_0_. This allows us to average the equations of motion for *a*_1,2_ over one mechanical period 2π/*Ω*_0_, resulting in

M1 $$\begin{array}{rc}{\dot{a}}_{j}=&\pm i\frac{{{\Delta }}{{\varOmega }}}{2}{a}_{j}-\frac{\gamma }{2}{a}_{j}+i\beta | {a}_{j}{| }^{2}{a}_{j}+\sqrt{\gamma {n}_{{\mathrm{T}}}}{\xi }_{j}(t)\\ &\pm i(\;{g}_{{\mathrm{r}}}\pm {g}_{{\mathrm{a}}})({a}_{2}-{a}_{1})\;f(2k{z}_{{{{\rm{zpf,0}}}}}| {a}_{2}-{a}_{1}| ),\end{array}$$

where the upper (lower) sign holds for *j* = 1(*j* = 2). Here, we define ${z}_{{{{\rm{zpf,0}}}}}=\sqrt{\hslash /2m{{{\varOmega }}}_{0}}$, the thermal occupation *n*_T_ = *k*_B_*T*_g_/*ℏΩ*_0_, the optical potential nonlinearity $\beta =3{{{\varOmega }}}_{0}{z}_{{{{\rm{zpf,0}}}}}^{2}/{z}_{{{{\rm{R}}}}}^{2}$, depending on the Rayleigh range *z*_R_ of the tweezers, and the reciprocal and anti-reciprocal coupling rates ${g}_{{\mathrm{r}}}=G\cos (k{d}_{0})\cos ({{\Delta }}\phi )/k{d}_{0}$ and ${g}_{{\mathrm{a}}}=G\sin (k{d}_{0})\sin ({{\Delta }}\phi )/k{d}_{0}$ as in the main text. They depend on the constant

M2 $$G=\frac{{\varepsilon }_{0}{\chi }^{2}{V}^{2}{k}^{5}{E}_{0}^{2}}{16\uppi m{{{\varOmega }}}_{0}}{\cos }^{2}\theta ,$$

with the particle volume *V* and electric susceptibilty *χ* = 3(*ε*_*r*_ − 1)/(*ε*_*r*_ + 2) with relative electric permeability *ε*_r_, the local tweezer field strength *E*_0_ and the angle (π/2 − *θ*) between tweezer polarization and particle-connecting axis. Importantly, the relevant nonlinearity in equations (3) and (M1) causing the limit cycle is not the optical potential nonlinearity *β*, but the nonlinearity in the dipole–dipole interaction described by the function *f*. As a result of the average over one mechanical cycle, (1) the non-conservative radiation pressure forces cancel, and (2) the gas diffusion noises *ξ*_*j*_(*t*) turn complex-valued.

#### First-order correlation functions

To obtain the linear model (1) for deeply trapped particles, we harmonically expand equation (M1) around *a*_1,2_ = 0, using *f*(0) = 1. Then, the autocorrelation functions ${g}_{jj}^{(1)}=\langle {a}_{j}^{* }(t){a}_{j}(t+{{\Delta }}t)\rangle$ for *j* ∈ {1, 2}, as well as the cross-correlation function ${g}_{12}^{(1)}=\langle {a}_{2}^{* }(t){a}_{1}(t+{{\Delta }}t)\rangle$ of deeply trapped particles follow from a straightforward calculation, yielding

M3 $$\begin{array}{rc}&{g}_{jj}^{(1)}({{\Delta }}t)={n}_{{\mathrm{T}}}\left[\left(1+\frac{4{g}_{{\mathrm{a}}}({g}_{{\mathrm{a}}}\pm {g}_{{\mathrm{r}}})}{{\gamma }^{2}+{\kappa }^{2}}\right)\cos \left(\frac{\kappa }{2}{{\Delta }}t\right)\right.\\ &+\frac{4\gamma {g}_{{\mathrm{a}}}({g}_{{\mathrm{a}}}\pm {g}_{{\mathrm{r}}})}{\kappa ({\gamma }^{2}+{\kappa }^{2})}\sin \left(\frac{\kappa }{2}| {{\Delta }}t| \right)\\ &\left.\pm i\frac{{{\Delta }}{{\varOmega }}-2{g}_{{\mathrm{a}}}}{\kappa }\sin \left(\frac{\kappa }{2}{{\Delta }}t\right)\right]{{\mathrm{e}}}^{-i{g}_{{\mathrm{r}}}{{\Delta }}t}{{\mathrm{e}}}^{-\frac{\gamma }{2}| {{\Delta }}t| },\end{array}$$

M4 $$\begin{array}{rc}&{g}_{12}^{(1)}({{\Delta }}t)={n}_{{\mathrm{T}}}\left[\frac{2{g}_{{\mathrm{a}}}(i\gamma +2{g}_{{\mathrm{a}}}-{{\Delta }}{{\varOmega }})}{{\gamma }^{2}+{\kappa }^{2}}\left\{\cos \left(\frac{\kappa }{2}{{\Delta }}t\right)\right.\right.\\ &\left.\left.+\frac{\gamma }{\kappa }\sin \left(\frac{\kappa }{2}| {{\Delta }}t| \right)\right\}+i\frac{2{g}_{{\mathrm{r}}}}{\kappa }\sin \left(\frac{\kappa }{2}{{\Delta }}t\right)\right]{{\mathrm{e}}}^{-i{g}_{{\mathrm{r}}}{{\Delta }}t}{{\mathrm{e}}}^{-\frac{\gamma }{2}| {{\Delta }}t| },\end{array}$$

with $\kappa =\sqrt{{{\Delta }}{{{\varOmega }}}^{2}-4{g}_{{\mathrm{a}}}{{\Delta }}{{\varOmega }}+{g}_{{\mathrm{r}}}^{2}}$. In the ${{{\mathcal{PT}}}}$ symmetry-broken regime, where *κ* becomes imaginary, the oscillatory behaviour in equation (M3) changes to a bi-exponential decay, in excellent agreement with the observed correlation functions (Fig. 4d,e).

#### Generalized ${{\boldsymbol{\mathcal{PT}}}}$ symmetry

The matrix *H*_NH_ exhibits a generalized version of ${{{\mathcal{PT}}}}$ symmetry, which is broken in the regime between the EPs. Ignoring gas damping for now, the matrix *H*_NH_ in equation (2) is symmetric under complex conjugation as ${H}_{{{{\rm{NH}}}}}^{* }={H}_{{{{\rm{NH}}}}}$. While *H*_NH_ is not invariant under the exchange of the two particles, it is symmetric under the simultaneous exchange of the particles and the tweezers trapping them. Mathematically, this is captured by the generalized transformations of the parity operator *P* = *U*^†^*σ*_*x*_*U*, where *σ*_*x*_ is the Pauli matrix and the unitary matrix

M5 $$U=\frac{1}{\sqrt{2}}\left(\begin{array}{rc}1&i\\ i&1\end{array}\right)$$

diagonalizes the anti-symmetric part of *H*_NH_, and the time reversal operator *T* = *U*^†^*CU*, where *C* denotes complex conjugation^1^. The matrix *H*_NH_ is then invariant under the generalized ${{{\mathcal{PT}}}}$ transformation as (*PT*)*H*_NH_(*PT*)^−1^.

#### Limit cycle model

The limit cycle model (3) follows from equation (M1) by ignoring gas diffusion, choosing the anti-reciprocal interaction as *g*_r_ = 0 and writing the complex amplitudes in terms of occupations and phases as ${a}_{1,2}=\sqrt{{n}_{1,2}}\exp (-i{\psi }_{1,2})$. It follows that the occupation difference *n*_2_ − *n*_1_ is always damped to zero on the timescale of the gas damping constant *γ*, while both occupations *n*_1,2_ and the mechanical phase delay *ψ* = *ψ*_2_ − *ψ*_1_ decouple from the mean mechanical phase (*ψ*_1_ + *ψ*_2_)/2. Assuming that *n*_2_ − *n*_1_ has already settled at zero, and defining the dimensionless effective oscillation amplitude by $A=2\sqrt{2}k{z}_{{{{\rm{zpf,0}}}}}\sqrt{{n}_{1}+{n}_{2}}$, we finally arrive at equation (3). Note that the optical potential nonlinearity *β* cancels from the effective limit cycle model (3), as its influence on the phase delay *ψ* vanishes if the particles oscillate with identical amplitude.

### Comparison of particle sizes

**Extended Data Fig. 1:**
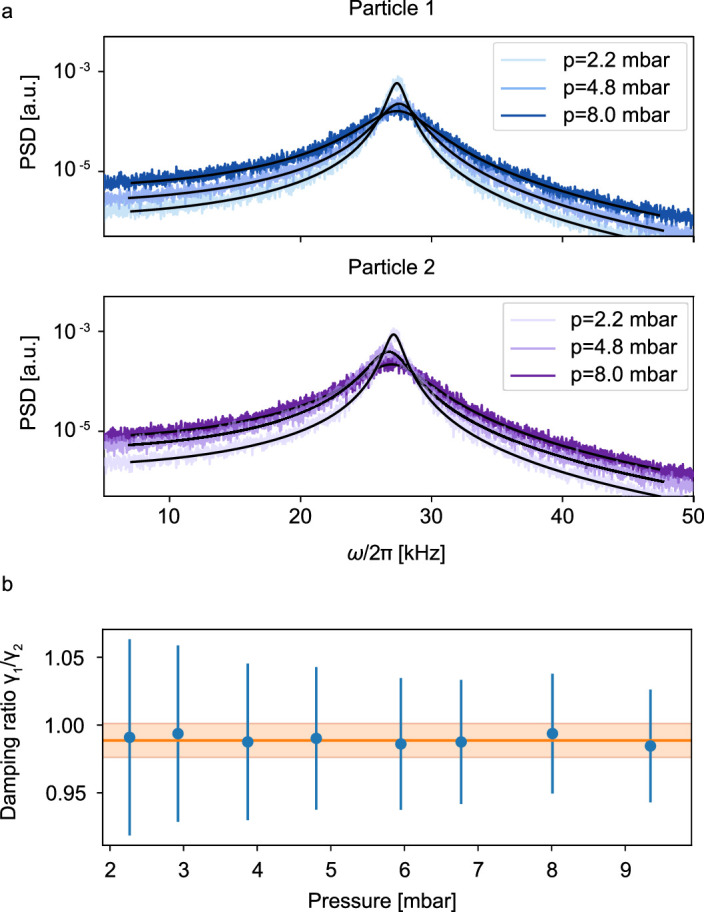
Ratios of particle damping rates at different pressures. **a**. Calculated particle PSDs at three different pressures for particles 1 (blue) and 2 (purple) illustrate how the damping rates *γ*_1,2_ decrease as the gas pressure decreases. The black lines correspond to the fitted lineshapes. **b**. Ratios of the fitted damping rates at different pressures (blue points and error bars) yields the weighted average (orange line) and its standard deviation (orange shaded region) of *γ*_1_/*γ*_2_ = 0.99 ± 0.01. The error bars are the propagated standard errors of the fits.

We assume identical spherical particles in size and mass in the theoretical models. The particles used in the experiment have a nominal radius of *r* = (105 ± 2) nm (microParticles GmbH); therefore, they could be of slightly different sizes. In the regime dominated by gas damping, the damping rate of the particle CoM motion depends on the particle radius as *γ* ∝ 1/*r* (ref. ^67^). Therefore, we measure and compare the damping rates of the two particles as a function of gas pressure to determine the ratio of the particle radii^43^. At each pressure, we calculate the power spectral densities (PSDs) of the CoM motion and extract the gas damping rates of particles 1 (blue) and 2 (purple) from the fit of the driven damped harmonic oscillator^68^ (Extended Data Fig. 1a). We assume equal mass densities and, due to the absence of rotational signatures in the PSDs, spherical particles. This relates the ratio of the damping rates of the two particles simply to the inverse ratio of the particle radii *γ*_1_/*γ*_2_ = *r*_2_/*r*_1_. We obtain the average ratio of the damping rates *γ*_1_/*γ*_2_ = 0.99 ± 0.01, and determine the size ratio to be *r*_1_/*r*_2_ = 1.01 ± 0.01 (Extended Data Fig. 1b). We conclude that the particles can be considered of approximately equal sizes.

### Calibration of the particle distance

**Extended Data Fig. 2:**
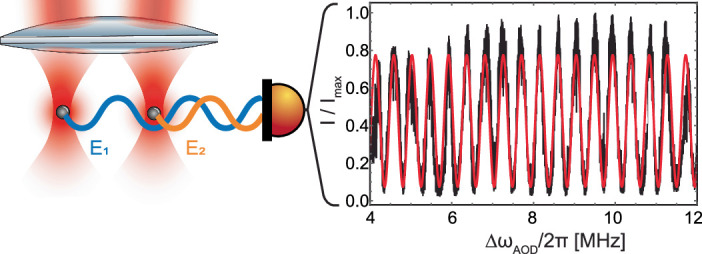
Distance calibration. The scattered light from particles 1 and 2 interferes in the far field. We measure the intensity of the interference pattern while scanning the particle distance (black line). The fringe spacing is given by the wavelength of the trapping light. We fit the pattern (red line) to determine the calibration constant from the frequency difference to particle distance.

The mean particle distance *d*_0_, given by the positions of the foci of their respective tweezers, influences the reciprocal and anti-reciprocal coupling rates. In our experiment, we tune the frequency difference Δ*ω* of the RF tones to modify the distance as *d*_0_ = *C*_0_Δ*ω*, where *C*_0_ is the conversion factor that can be calibrated by comparing the distance to a known etalon, for example, the laser wavelength. Therefore, we perform an interferometric measurement of the dipole radiation from the two particles with a detector placed in the far field, along the axis connecting the particles (Extended Data Fig. 2). The tweezer polarizations are set perpendicular to this axis, such that the dipole radiation in the direction of the detector is maximal. As the images of the two particles are indistinguishable at the detector, the overlap of the dipole radiations leads to an interference that depends on *d*_0_. In our measurement, we sweep Δ*ω*/2π in the range of 4–12 MHz and record the intensity of the interference pattern. We note that the magnitude of the interference pattern changes as a function of Δ*ω*. As the measurement is fully repeatable, we attribute this to the varying AOD diffraction efficiencies as a function of the RF tone frequencies. We fit the data with a sine function and obtain the conversion factor *C*_0_ = (2.3845 ± 0.0005) μm MHz^−1^. All measurements in the main text were performed at a frequency difference of Δ*ω*/2π = 7.72 MHz, which leads to an estimated distance of *d*_0_ = (18.408 ± 0.005) μm.

### Detection of the particle motion

**Extended Data Fig. 3:**
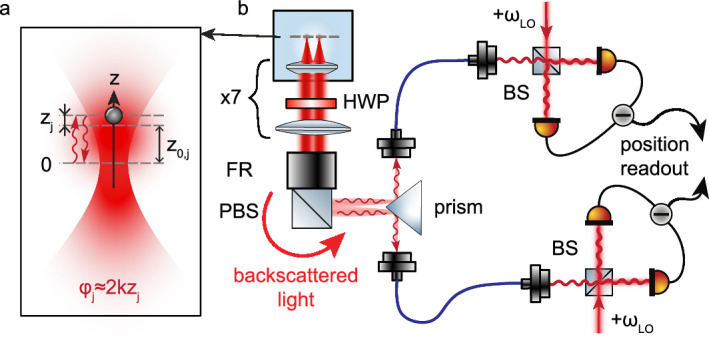
Detection of the particles’ motion. **a**. A particle is trapped in a tightly focused Gaussian beam at a position *z*_0,*j*_ away from the focus. The phase of the backscattered light changes with the particle motion *z*_*j*_ around the trap position as *φ*_*j*_ ≈ 2*kz*_*j*_. **b**. Sketch of the balanced heterodyne detection scheme. The backscattered light is separated from the incoming beams by a Faraday rotator (FR) and a polarizing beamsplitter (PBS) and subsequently reimaged onto a prism mirror to spatially separate the light scattered by the two particles. Each particle’s image is collected with a single-mode fiber in a confocal microscope configuration for further spatial filtering from the background reflections. The particles’ signals are combined with a local oscillator at a frequency of *ω*_LO_ with a 50:50 beamsplitter and are measured with balanced photodetectors.

The particles scatter light and imprint the information about their position onto the phase of the scattered light^69^. In our case, the optical phase of the back-scattered light optimally maps the particles’ motion along the optical axis (Extended Data Fig. 3a). The optical phase *φ*_*j*_ can be approximated as the sum of contributions from the three-dimensional motion:

M6 $${\varphi }_{j}({r}_{j}(t))={\varphi }_{j}({z}_{j})+{\varphi }_{j}(\;{y}_{j})+{\varphi }_{j}({x}_{j}).$$

However, the *z* motion is well separated in frequency from the *x* and *y* motion owing to the trap asymmetry (Ω_*z*,*j*_ ≪ Ω_*x*,*j*_, Ω_*y*,*j*_). Therefore, we can isolate the *z* motion contribution by applying a bandpass filter with the bandwidth (5, 50) kHz around the average *z* frequency Ω_0_/2π = 27.5 kHz. Furthermore, the transverse motion influences the optical phase around 100 times less than *z*_*j*_ in the backplane detection.

To split the back-scattered light from the trapping light, we use a polarizing beamsplitter and a Faraday rotator (Extended Data Fig. 3b). The image of the particle plane is magnified 160-fold onto a prism mirror that separates the individual particle images into opposite directions. The split particle signals are collected with two single-mode fibres such that the confocal microscope magnification (×7) between the trapping plane and the fibre input plane maximizes the collection efficiency^70,71^. The collected scattered light (optical power of 5–10 μW) is mixed on a beamsplitter with a reference beam (local oscillator) (optical power of 1–2 mW, frequency-shifted by *ω*_LO_/2π = 1.1 MHz) to implement a balanced heterodyne detection.

The heterodyne signal to the leading order in the particle motion *z*_*j*_ yields

M7 $${I}_{-,\;j}(t)\propto \left\vert {E}_{{\mathrm{LO}}}^{* }{E}_{{\mathrm{sc}}}\right\vert \cos \left({\omega }_{{\mathrm{LO}}}t+{\varphi }_{j}[{z}_{j}(t)]\right),$$

where *E*_LO_ and *E*_sc_ are the strengths of the local oscillator and scattered fields, respectively. We retrieve the optical phases *φ*_*j*_ by calculating the Hilbert transform of *I*_−,*j*_, which allows us to obtain the unwrapped argument of *I*_−,*j*_. Finally, we subtract the known accumulated phase of the local oscillator to obtain

M8 $${\varphi }_{j}({z}_{j}(t))={{{\rm{unwr}}}}\left\{\arg \left({{{\mathcal{H}}}}\left[{I}_{-,\;j}(t)\right]\right)\right\}-{\omega }_{{\mathrm{LO}}}t.$$

The obtained phase can now be converted into the position *z*_*j*_ as^69^

M9 $${\varphi }_{j}({z}_{j})=2k{z}_{j}-\arctan \left(({z}_{j}+{z}_{0,\;j})/{z}_{{{{\rm{R}}}}}\right),$$

where $\arctan \left(({z}_{j}+{z}_{0,\;j})/{z}_{{{{\rm{R}}}}}\right)$ is the modification due to the Gouy phase with the constant offset from the focus *z*_0,*j*_, the Rayleigh length *z*_R_ = *w*_*x*_*w*_*y*_π/*λ* and waists *w*_*x*_ and *w*_*y*_ along the *x* axis and *y* axis, respectively. We numerically calculate the tweezer waists *w*_*x*_ = 0.678 μm and *w*_*y*_ = 0.775 μm from the measured mechanical frequencies and *z*_0,*j*_ = 0.8 μm from the particle radius to estimate the correction due to the Gouy phase. The obtained Rayleigh length of *z*_R_ = 1.55 μm results in the modification of ~3% to the particle motion detection. Therefore, we safely omit this correction and use *φ*_*j*_(*z*_*j*_) = 2*kz*_*j*_ instead in the main text.

### Measurement of the coupling rates

In this section, we detail the experimental methods to optimize the anti-reciprocal coupling rate *g*_a_ and to reduce the reciprocal coupling rate *g*_r_ and the coupling rate *g*_C_ due to electrostatic interactions.

#### Electrostatic coupling

**Extended Data Fig. 4:**
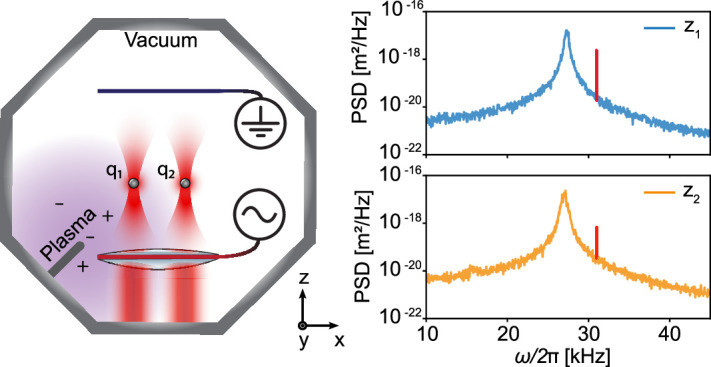
Charge control and calibration. To change the amount of net charges attached to the particles we ignite a plasma at a high-voltage pin inside the vacuum chamber. We monitor the number of charges by driving the particles via an AC field, applied to electrodes mounted to the trapping lens (signal, red line) and a lens tube (ground, dark blue line), at a relative distance of *D*_*e*_ = (5 ± 1) mm. The electric drive can be seen as a sharp peak (red) in the power spectral densities if the particles are charged. We estimate the remaining charges to be *q*_1_ = (2.5 ± 0.7) *e* and *q*_2_ = (1.5 ± 0.5) *e*, where *e* is the elementary charge.

To minimize the electrostatic interaction, we discharge the particles by igniting a plasma at a high-voltage pin inside the vacuum chamber (Extended Data Fig. 4a). We attach wires to the holders of the trapping lens and the lens that collects light after the tweezer foci, which forms a capacitor for the charged particle with holders acting as the electrodes. We continuously drive the particle motion with a sinusoidal signal applied to two electrodes, which yields a sharp peak at the drive frequency in the PSD of the particle motion. When plasma is ignited, the number of charges on each particle fluctuates, which we monitor as changes in the peak amplitude in real time. We switch off the high voltage when both particles have just a few charges, which is reflected in low amplitudes of the drive peak in the PSD.

To estimate the electrostatic interaction after the discharging procedure, we follow the procedure in the supplementary information of ref. ^41^. To calibrate the number of charges on each particle, we drive the motion of both particles by applying a sinusoidal voltage with an amplitude of *V*_AC_ = 10 V and at a frequency *Ω*_*d*_/2π = 31 kHz to the electrodes and record the particle motion. Assuming a model of a point charge in a plate capacitor, we estimate the number of charges *N*_1,2_ to be

M10 $${N}_{1,2}=\frac{\sqrt{2}m{D}_{{\mathrm{e}}}}{e{V}_{{\mathrm{AC}}}}\left\vert {{{\varOmega }}}_{1,2}^{2}-{{{\varOmega }}}_{{{d}}}^{2}\right\vert \sqrt{\left\langle {z}_{{{d}}1,2}^{2}\right\rangle },$$

where *m* is the particle mass, *D*_e_ = (5 ± 1) mm is the distance between the electrodes, *e* is the elementary charge, *Ω*_1,2_ are the oscillation frequencies of particles 1 and 2, and $\langle {z}_{{{d}}1,2}^{2}\rangle$ is the time-averaged displacement of particles 1 and 2 along the *z* axis due to the electronic drive. The PSDs of the driven motion can be seen in Extended Data Fig. 4b. The peak due to the electric drive is marked in red. We estimate the number of charges to be *N*_1_ = 2.5 ± 0.7 and *N*_2_ = 1.5 ± 0.5, which gives an electrostatic coupling rate of

M11 $${g}_{{\mathrm{C}}}=-\frac{{N}_{1}{N}_{2}{e}^{2}}{8\uppi {\varepsilon }_{0}m{{\varOmega }}^{\prime} {d}_{0}^{3}}=(-0.077\pm 0.030)\,{{{\rm{Hz}}}}.$$

Here, *ε*_0_ is the vacuum permittivity and ${{\varOmega }}^{\prime} =\sqrt{{{{\varOmega }}}^{2}-{N}_{1}{N}_{2}{e}^{2}/(4\uppi {\varepsilon }_{0}m{d}_{0}^{3})}$. This coupling rate is more than two orders of magnitude smaller than the intrinsic damping rate and the measured anti-reciprocal coupling rates for polarization angles in the range (0°, 80°). Therefore, the electrostatic interaction does not affect the non-Hermitian dynamics in our experiment.

#### Optical coupling

The optical coupling rates *g*_r_ and *g*_a_ depend on the particle distance *d*_0_ and the optical phase difference Δ*ϕ*. To maximize *g*_a_ and minimize *g*_r_, we scan both parameters while the trap polarization is set to ~0° to maximize the optical interaction. For a general combination of parameters where *g*_a_ ≠ 0 and *g*_r_ ≠ 0, the total coupling rates *g*_r_ ± *g*_a_ are different in opposite directions. In this case, the response to the motion of particle 2 in the PSD of particle 1, with a peak amplitude $\propto {({g}_{{\mathrm{r}}}+{g}_{{\mathrm{a}}})}^{2}$ at frequency *Ω*_2_ in the PSD, is different from the peak amplitude $\propto {({g}_{{\mathrm{r}}}-{g}_{{\mathrm{a}}})}^{2}$ of the particle 1 at frequency *Ω*_1_ in the PSD of particle 2. For any combination of *d*_0_ and Δ*ϕ*, we monitor the PSD of both particles and we choose the parameters such that the two contributions have matching amplitudes, which is satisfied either for *g*_r_ = 0 or *g*_a_ = 0. As the final step, we make sure that there is no normal mode splitting but normal mode attraction to confirm that *g*_r_ ≡ 0. For such a configuration, we estimate that Δ*ϕ* = (0.5 ± 0.08)π owing to the step size of the scan. With the previously estimated distance of *d*_0_ = (18.408 ± 0.005) μm, the reciprocal coupling ${g}_{{\mathrm{r}}}\propto \cos (k{d}_{0})\cos ({{\Delta }}\phi )=0.00\pm 0.08$ is effectively zero.

**Extended Data Fig. 5:**
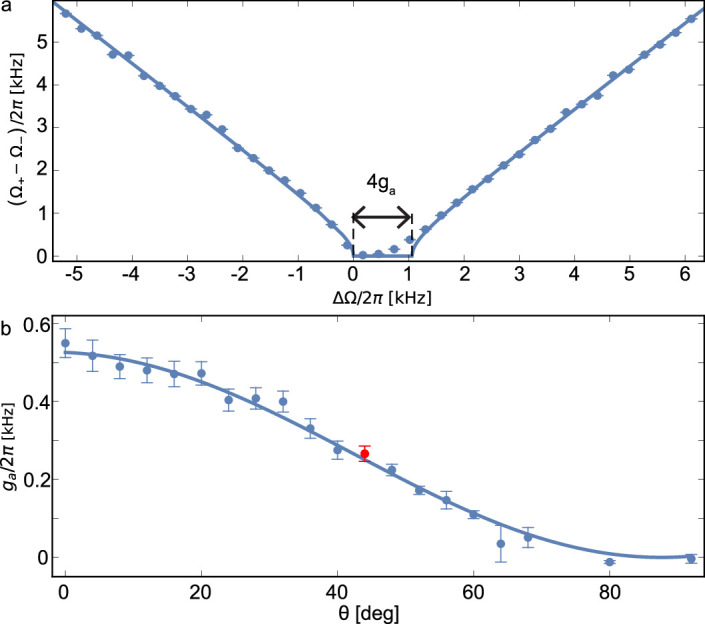
Anti-reciprocal coupling rate. **a**. We fit the eigenfrequency difference Ω_+_ − Ω_−_ as a function of the intrinsic frequency detuning ΔΩ with linear theory (line) to extract the coupling rate *g*_*a*_ for each polarization *θ*. Points are obtained from fitting spectral peaks. Error bars represent the standard error of the fit. **b**. The anti-reciprocal coupling rate follows a ${\cos }^{2}\theta$ dependence of the tweezer polarization angle *θ*. The red point represents the detuning scan in **a**. Points represent the values obtained form the fit while error bars represent the standard error of the fit.

To determine the magnitude of the anti-reciprocal coupling rate *g*_a_, we fit the difference of the eigenfrequencies ${{{\varOmega }}}_{+}-{{{\varOmega }}}_{-}={\mathrm{R}}{\mathrm{e}} (\sqrt{{{\Delta }}{{{\varOmega }}}^{2}-4{g}_{{\mathrm{a}}}{{\Delta }}{{\varOmega }}})$, obtained from the PSDs, as a function of the intrinsic frequency detuning Δ*Ω* (Extended Data Fig. 5a). We attribute the non-zero values in the ${{{\mathcal{PT}}}}$ symmetry-broken phase to small differences in the particle masses and the thermal fluctuations, which are not accounted for in the linear model of the eigenfrequencies. We determine *g*_a_ as a function of the tweezer polarization *θ* (Extended Data Fig. 5b), which follows a ${\cos }^{2}\theta$ function, as predicted from the light-induced dipole–dipole interactions.

### Evaluation of the phase delay and the displacement amplitude

#### Phase delay

To compute the oscillation phases ${\bar{\psi }}_{1,2}$, we first apply the Hilbert transform to the individual particle motion *z*_1,2_ to obtain the complex amplitude ${\tilde{z}}_{1,2}(t)={{{\mathcal{H}}}}\left[{z}_{1,2}\right]$ such that ${z}_{1,2}(t)={\mathrm{R}}{\mathrm{e}} ({\tilde{z}}_{1,2}(t))$. The oscillation phases are recovered from the unwrapped phases of the complex amplitudes:

M12 $${\bar{\psi }}_{j}({z}_{j}(t))={{{\rm{unwr}}}}\left\{\arg \left({{{\mathcal{H}}}}\left[{z}_{j}(t)\right]\right)\right\}.$$

We define the instantaneous phase delay between the oscillators as $\bar{\psi }={\bar{\psi }}_{2}-{\bar{\psi }}_{1}$. The interaction generates a preferred phase delay ${\bar{\psi }}_{{\mathrm{max}}}$ as observed in the histograms of $\bar{\psi }$, while it is uniformly distributed in the case of uncoupled oscillators (Fig. 2b).

#### Displacement amplitude

**Extended Data Fig. 6:**
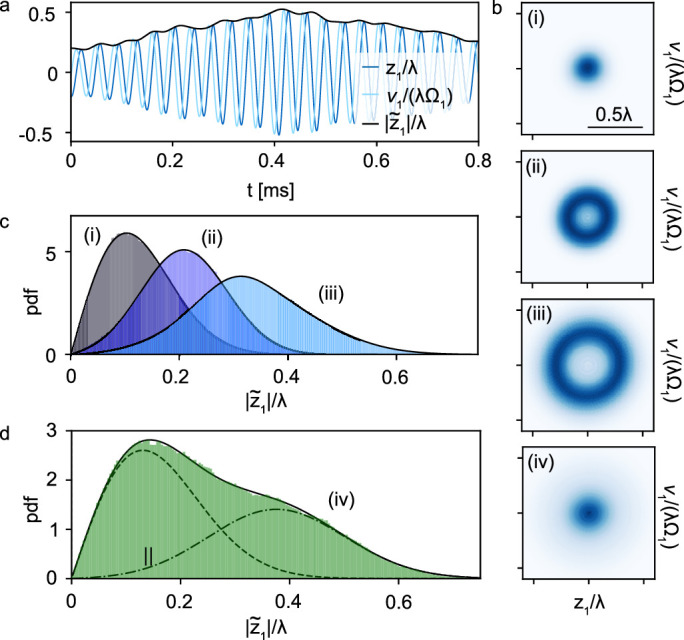
Evaluation of the displacement amplitude. **a**. Short time trace of the particle motion (blue), velocity (light blue), and the reconstructed oscillation envelope (black). **b**. Phase space distributions of the motion of particle 1 sampled over the whole time trace for four different configurations, labeled as (i)-(iv). The limit cycle is observed in (ii) and (iii), while no limit cycle is observed in (iv). **c**. The probability distribution functions (pdf) of the oscillation envelopes for configurations (i)-(iii). Solid black lines represent the fits of the Rice distribution, where (ii) and (iii) clearly show the increase of the displaced amplitude ${\bar{A}}_{1}$ from the case of ${\bar{A}}_{1}=0$ in (i). The presented experimental data corresponds to the following detuning and coupling rate (ΔΩ/2*π*, *g*_*a*_/2*π*) and extracted displacement amplitudes ${\bar{A}}_{1}$: (i) (4.78, 0.00) kHz and 0, (ii) (0.40, 0.28) kHz and 1.7, (iii) (0.35, 0.49) kHz and 2.7. **d**. Histogram of ${\tilde{z}}_{1}/\lambda$ in the vicinity of the NIR region for (ΔΩ/2*π*, *g*_*a*_/2*π*) = ( − 0.22, 0.49) kHz, corresponding to the distribution (iv), exhibits two peaks. The solid black line is the bi-modal Rice distribution fit, while the dashed and dash-dotted lines show the contributions of two Rice distributions with fitted ${\bar{A}}_{1}=0$ and ${\bar{A}}_{1}=3.1$, respectively.

The nonlinear interaction regime (NIR) arises for the negative effective damping *γ*_−_ < 0 as predicted by the linearized theory. This results in the modified statistics of the particles’ motion and the emergence of a limit cycle (Fig. 2a). To analyse this regime quantitatively and to compare the results to the theoretical model (3), we calculate the histograms of the oscillation envelopes $| {\tilde{z}}_{j}|$ for each particle and fit them with a model accounting for the possible emergence of a limit cycle with a displacement amplitude ${\bar{A}}_{j}$ (Extended Data Fig. 6a)^72^.

Outside the NIR, there is no limit cycle; therefore, ${\bar{A}}_{j}=0$. There, the positions (as well as velocities) assume Gaussian distributions centred around zero with a width *σ* that depends on the effective thermal occupation. The histograms of $| {\tilde{z}}_{j}(t)|$ are described by the Rayleigh distribution ${P}_{{{{\rm{Ray}}}}}(x,\sigma )=(x/{\sigma }^{2})\exp (-{x}^{2}/2{\sigma }^{2})$ as a function of the position *x*. As soon as the limit cycle trajectories emerge, the displaced amplitude ${\bar{A}}_{j}$ becomes non-zero. There, the oscillation envelope follows a Rice distribution that generalizes the Rayleigh distribution for the case of non-centred Gaussian random variables. The Rice distribution has previously been used for the statistical description of pre-condensed light^73^. In our case, it is defined as

M13 $${P}_{{{{\rm{Rice}}}}}\left(x,\sigma ,{\bar{A}}_{j}\right)=\frac{x}{{\sigma }^{2}}{{\mathrm{e}}}^{-\frac{{x}^{2}+{\bar{A}}_{j}^{2}}{2{\sigma }^{2}}}{{{{\mathcal{I}}}}}_{0}\left(\frac{x{\bar{A}}_{j}}{{\sigma }^{2}}\right),$$

where ${{{{\mathcal{I}}}}}_{0}$ is the modified Bessel function of the first kind with order zero. Note that the thermal (coherent) statistics are recovered from the Rice distribution in the limit of ${\bar{A}}_{j}\to 0$ (${\bar{A}}_{j}\gg \sqrt{\sigma }$).

We fit the Rice distribution to the histograms of the envelope $| {\tilde{z}}_{j}|$ with ${\bar{A}}_{j}$ and *σ* as free parameters. The fitted amplitudes are then used to calculate the collective displaced amplitude $\bar{A}=2k\sqrt{\hslash /(m{{{\varOmega }}}_{0})}\sqrt{{\bar{A}}_{1}^{2}+{\bar{A}}_{2}^{2}}$ and compared with the theoretical amplitude *A* from equation (3). Extended Data Fig. 6c shows the histogram of the oscillation envelope for particle 1 fitted with a Rice distribution outside the NIR ((i)) as well as within the NIR for increasing interaction strength ((ii) and (iii)). In Extended Data Fig. 6b, we plot the corresponding position–velocity distributions of particle 1. In the vicinity of the NIR region, we observe a bimodal distribution (Extended Data Fig. 6d), which can be interpreted as a time average of the collective state occasionally jumping between the linear and nonlinear states. We attribute that to the thermal fluctuations-induced drifts of the mechanical frequencies around their average values. As a result, if the average frequencies are at the boundary of the limit cycle region, the particles spend some time in the limit cycle phase (${\bar{A}}_{j} > 0$) and the rest in the correlated thermal state (${\bar{A}}_{j}=0$) if otherwise. In these cases, we fit the histograms of the oscillation envelopes with a weighted sum of two different Rice distributions:

M14 $$\begin{array}{rcl}{P}_{j}(x)&=&\alpha {P}_{{{{\rm{Rice}}}}}\left(x,{\sigma }_{j,1},{\bar{A}}_{j,1}\right)\\ &+&(1-\alpha ){P}_{{{{\rm{Rice}}}}}\left(x,{\sigma }_{j,2},{\bar{A}}_{j,2}\right).\end{array}$$

For example, at a detuning of Δ*Ω*/2π = (−0.22 ± 0.15) kHz and a coupling rate of *g*_a_/2π = (0.49 ± 0.03) kHz, we obtain the amplitudes ${\bar{A}}_{1,1}=0$ with the weight *α* = 0.56 and ${\bar{A}}_{1,2}=3.1$ with the weight 1 − *α* = 0.44, thus confirming that our model explains the average collective dynamics well. In the case of a bimodal distribution for the individual oscillation envelope, we use the fitted displaced amplitude with the highest weight for the calculation of the collective displaced amplitude, shown in Fig. 4 of the main text.

### Experimental evaluation of the correlations

**Extended Data Fig. 7:**
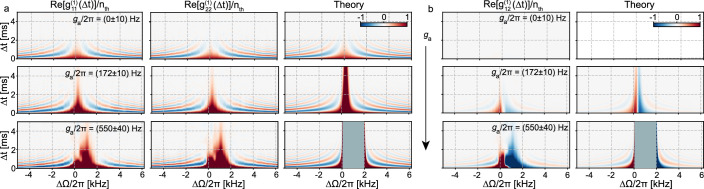
First-order correlations: comparison between the experiment and theory. Correlation functions are plotted as a function of the mechanical detuning and the time delay for increasing coupling rate *g*_*a*_ from top to bottom. **a**. The autocorrelation in the left and middle columns corresponds to the experimental data from particles 1 and 2, respectively, and the right column corresponds to the theoretical model. **b**. The experimental and theoretical crosscorrelations are shown in the left and right columns, respectively.

The time traces of the complex envelopes *a*_1/2_ were used to calculate the first-order correlations ${g}_{jj}^{(1)}/{n}_{{\mathrm{T}}}=\langle {a}_{j}^{* }(t){a}_{j}(t+{{\Delta }}t)\rangle /{n}_{{\mathrm{T}}}$ for *j* ∈ {1, 2}, and ${g}_{12}^{(1)}/{n}_{{\mathrm{T}}}=\langle {a}_{2}^{* }(t){a}_{1}(t+{{\Delta }}t)\rangle /{n}_{{\mathrm{T}}}$ by using Python’s ‘scipy.signal.correlate’ function. In Extended Data Fig. 7a, we show the measured autocorrelation functions of particles 1 and 2 (left and middle column) compared with the theoretical model (M3) (right column) for increasing coupling rates *g*_a_ from top to bottom. The autocorrelation is highest at zero time delay, where it corresponds to the motion variance. In the oscillatory phase, it exhibits decaying oscillations as a function of the time delay with a decay rate of half of the damping rate, in excellent agreement with the theory. The oscillation frequency depends on the intrinsic detuning; therefore, for uncoupled particles (upper row), the autocorrelations show an oscillating decay everywhere except at zero detuning. The variances increase with the coupling rate in the ${{{\mathcal{PT}}}}$-symmetry-broken phase and eventually lead to the limit cycle phase, where the linearized theory is not valid anymore (grey region in the theory plot, bottom row). Note that the autocorrelations look very similar for both particles, confirming the negligible contribution of the reciprocal coupling component (*g*_r_ ≈ 0), according to the equation (M3). The cross-correlation colour plots as a function of the mechanical detuning and the time delay are compared with the linearized model (M4) for increasing coupling rates from top to bottom in Extended Data Fig. 7b. The magnitude of the cross-correlation increases with the coupling rate for both theory and experiment and is maximal at Δ*t* = 0, where it corresponds to the amplitude covariance. Within the ${{{\mathcal{PT}}}}$ symmetry-broken regime, the sign of the correlations changes due to the change in the relative phase delay between the oscillations of the two particles.

## Online content

Any methods, additional references, Nature Portfolio reporting summaries, source data, extended data, supplementary information, acknowledgements, peer review information; details of author contributions and competing interests; and statements of data and code availability are available at 10.1038/s41567-024-02589-8.

## Data Availability

Source data for Figs. 1–4 and Extended Data Figs 1, 2 and 4–7 are available at https://phaidra.univie.ac.at/o:2068446. All other data that support the conclusions of this work are available from the corresponding author upon reasonable request.
